# Crystal structure of poly[μ_3_-acetato-di­aqua-μ_3_-sulfato-cerium(III)]: serendipitous synthesis of a layered coordination polymer exhibiting inter­layer O—H⋯O hydrogen bonding

**DOI:** 10.1107/S2056989024012313

**Published:** 2025-01-10

**Authors:** Niklas Ruser, Christian Näther, Norbert Stock

**Affiliations:** a Institute of Inorganic Chemistry, Kiel University, Max-Eyth-Str. 2, 24118 Kiel, Germany; University of Aberdeen, United Kingdom

**Keywords:** synthesis, crystal structure, solvothermal reaction, coordination polymer

## Abstract

Single crystals of the title compound, [Ce(CH_3_COO)(SO_4_)(H_2_O)_2_]_*n*_, were obtained serendipitously by the reaction of Ce(NO_3_)_3_·6H_2_O with 2,5-thio­phenedi­carb­oxy­lic acid (H_2_TDC) and acetic acid in a mixture of ethanol and water, where it is assumed that the sulfate ions leached from the Teflon reactor, which was treated with per­oxy­monosulfuric acid prior to its use. Its asymmetric unit consists of one Ce^III^ cation, one sulfate dianion, one acetate anion and two crystallographically independent water mol­ecules, all of them located in general positions. The cerium cations are coordinated by three acetate anions (one *O*,*O*-chelating) and three sulfate dianions that are related by symmetry as well as two independent water mol­ecules within an irregular CeO_9_ coordination geometry. The Ce^III^ cations are linked by the acetate ions into [010] chains, which are further connected into (001) layers by the sulfate dianions. Intra­layer and inter­layer O—H⋯O hydrogen bonds are observed. Powder X-ray diffraction shows that only traces of the title compound have formed together with a large amount of an unknown crystalline phase. Attempts to prepare the title compound in larger amounts and as a pure phase were unsuccessful.

## Abstract 2

In the crystal structure of the title compound, the Ce^III^ cations are ninefold coordinated and linked into chains *via* the acetate anions and are further connected into layers by the sulfate dianions.

## 1. Chemical context

In the search for new coordination polymers (CPs) (Batten *et al.*, 2009 ▸) or metal–organic frameworks (MOFs) (Rowsell & Yaghi, 2004 ▸; Long & Yaghi, 2009 ▸), many inorganic and organic building blocks have been used to construct such materials. Consisting of repeating units of metal atoms or ions bridged by coordinating ligands, the resulting frameworks of CPs extend in up to three dimensions. MOFs, on the other hand, are a subclass of CPs and contain only organic ligands, called linkers, and extend in two or three dimensions. Another requirement is the presence of potential pores (Batten *et al.*, 2013 ▸), which generate large specific surface areas that can be used for applications such as catalysis (Hu *et al.*, 2018 ▸; Li, 2018 ▸; Lammert *et al.*, 2015 ▸), gas storage (Li *et al.*, 2019 ▸; Sahayaraj *et al.*, 2023 ▸) and sensing (Shekhah *et al.*, 2011 ▸; Wang *et al.*, 2018 ▸). Depending on the metal ions and organic linkers used, the properties of MOFs can often be tailored (Sahayaraj *et al.*, 2023 ▸). For example, by using a metal such as cerium, its ability to change its oxidation state between +III and +IV can be exploited in catalysis (Lammert *et al.*, 2015 ▸; Smolders *et al.*, 2018 ▸, 2020 ▸).

There are as many different building blocks as there are reaction conditions to synthesize such materials. This leads to multidimensional parameter spaces that can be explored with many potential compounds to be discovered. The so-called high-throughput method is very useful when it comes to screening parameter spaces (Stock, 2010 ▸). With this method, many different syntheses can be carried out simultaneously while varying the reaction conditions. Some areas of a parameter space lead exclusively to one compound, *i.e.* phase pure compounds, while in other cases phase mixtures are observed. This work reports the synthesis and structure of the title Ce^III^ -CP discovered in a screening experiment by reacting equimolar amounts of a cerium nitrate and 2,5-thio­phenedi­carb­oxy­lic acid (H_2_TDC) and varying the ratio of acetic acid and ethanol/water mixture. Surprisingly, single crystals of a product were obtained that did not contain TDC^2–^ dianions, but rather sulfate dianions.
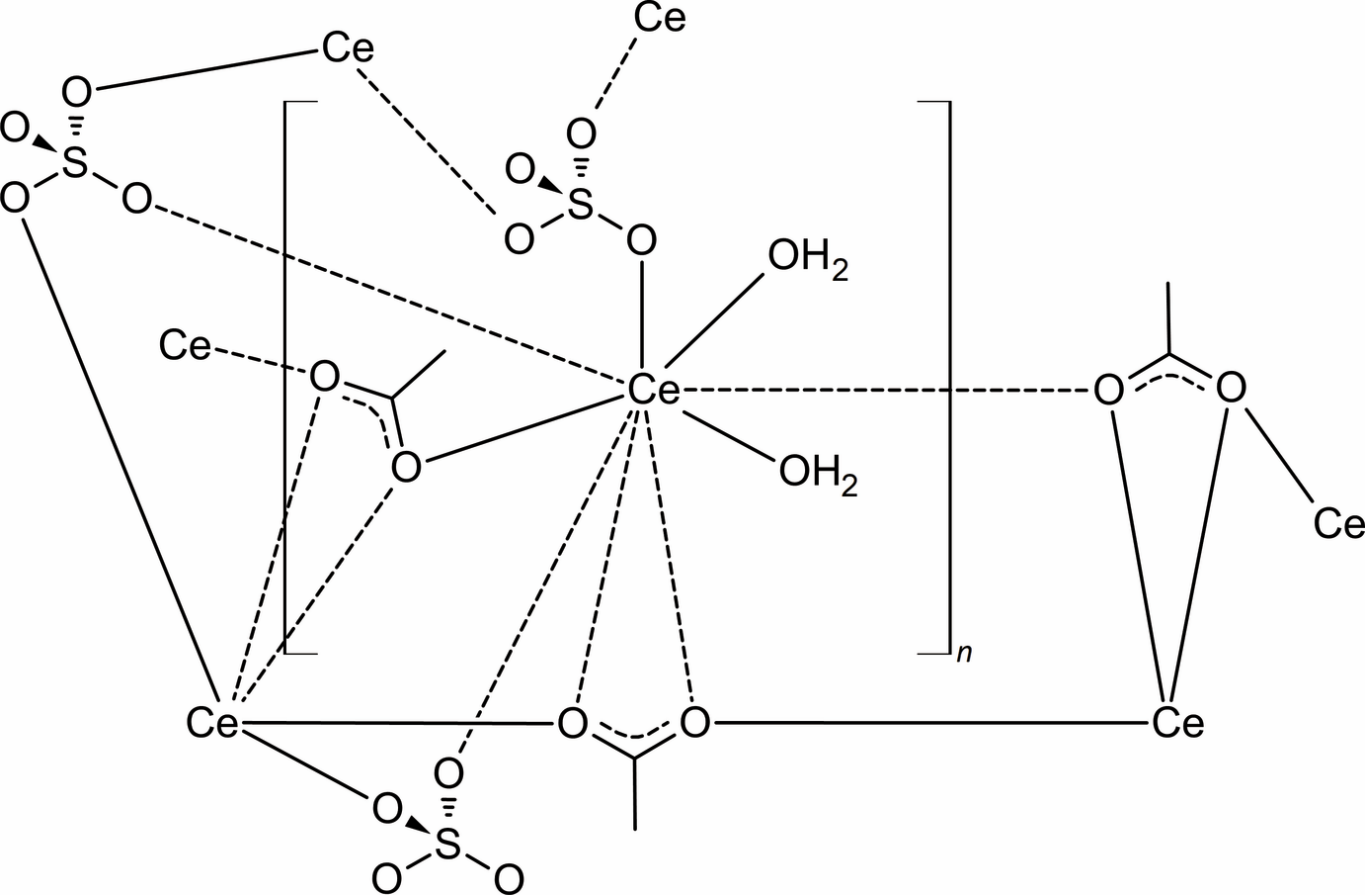


## 2. Structural commentary

**Figure 1:**
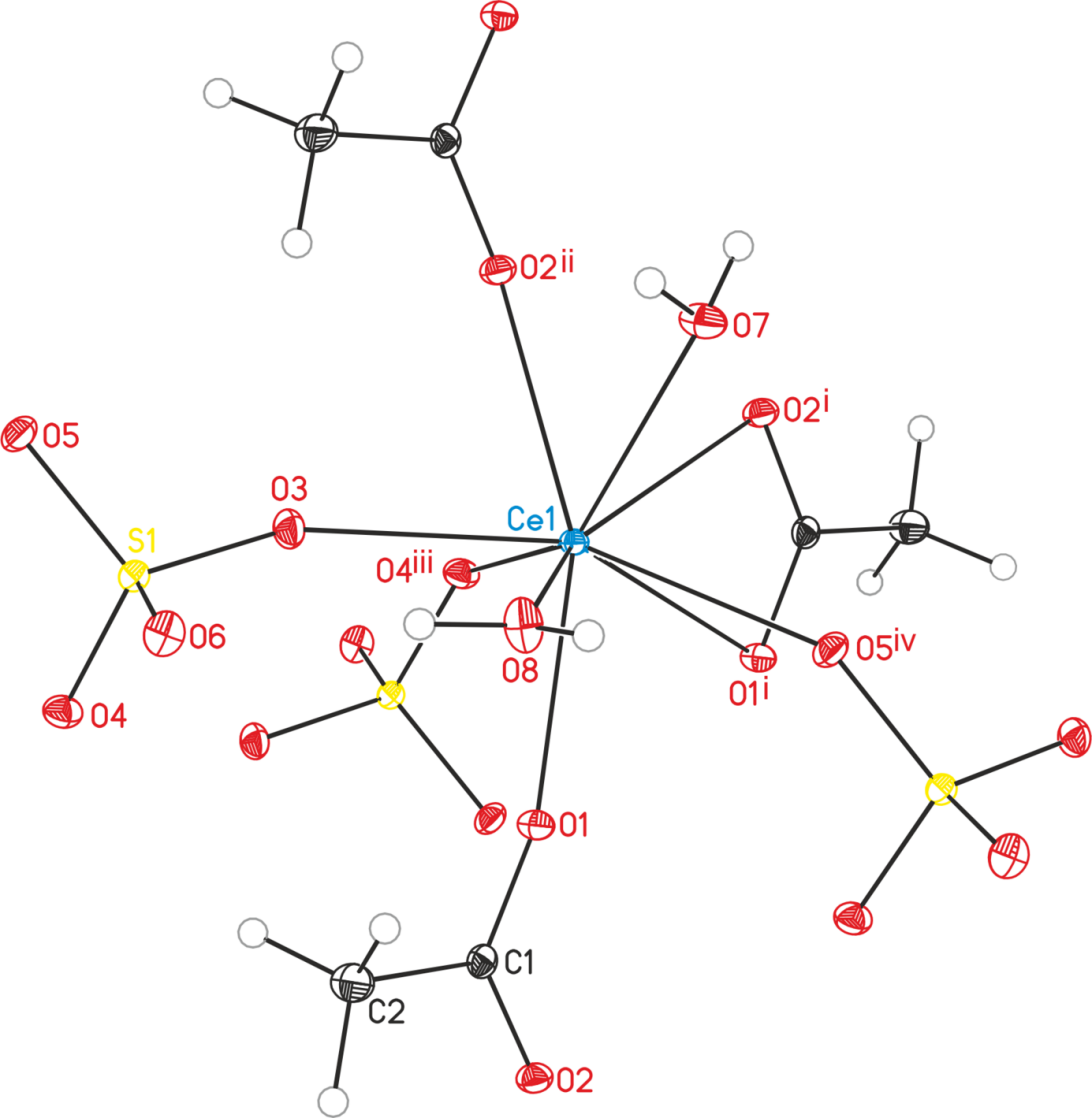
Crystal structure of the title compound with labelling and displacement ellipsoids drawn at the 50% probability level. Symmetry codes: (i) −*x*, −*y* + 1, −*z* + 1; (ii) *x*, *y* − 1, *z*; (iii) −*x* + 1, −*y* + 1, −*z* + 1; (iv) *x * − 1, *y*, *z*.

**Figure 2:**
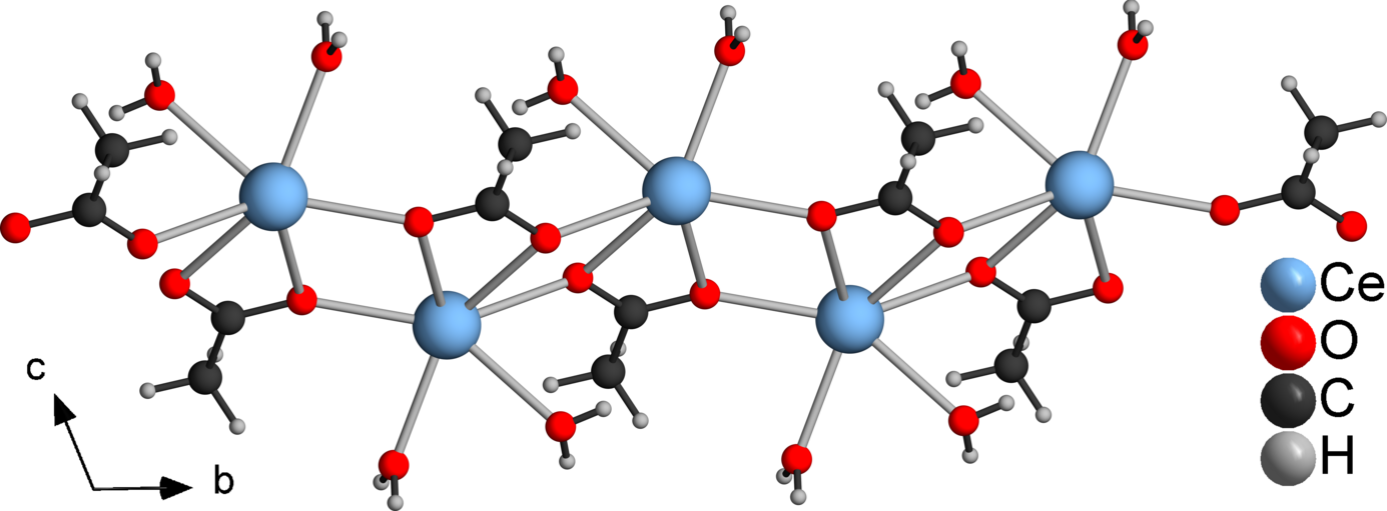
Crystal structure of the title compound showing a section of a cerium–acetate chain.

**Figure 3:**
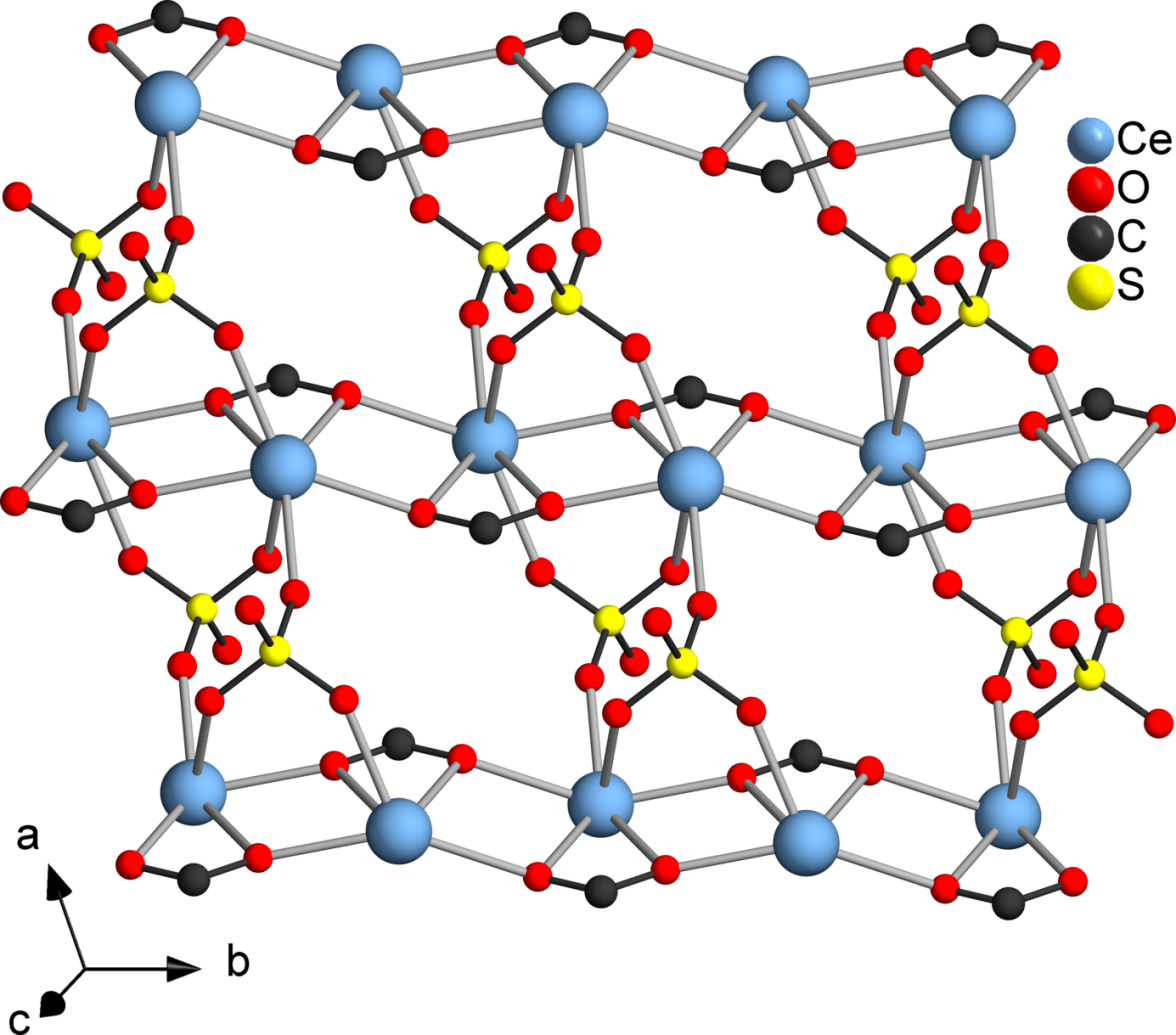
Connection of the cerium–acetate chains by sulfate dianions into layers. The methyl groups of the acetate anions and the water mol­ecules were omitted for clarity.

**Figure 4:**
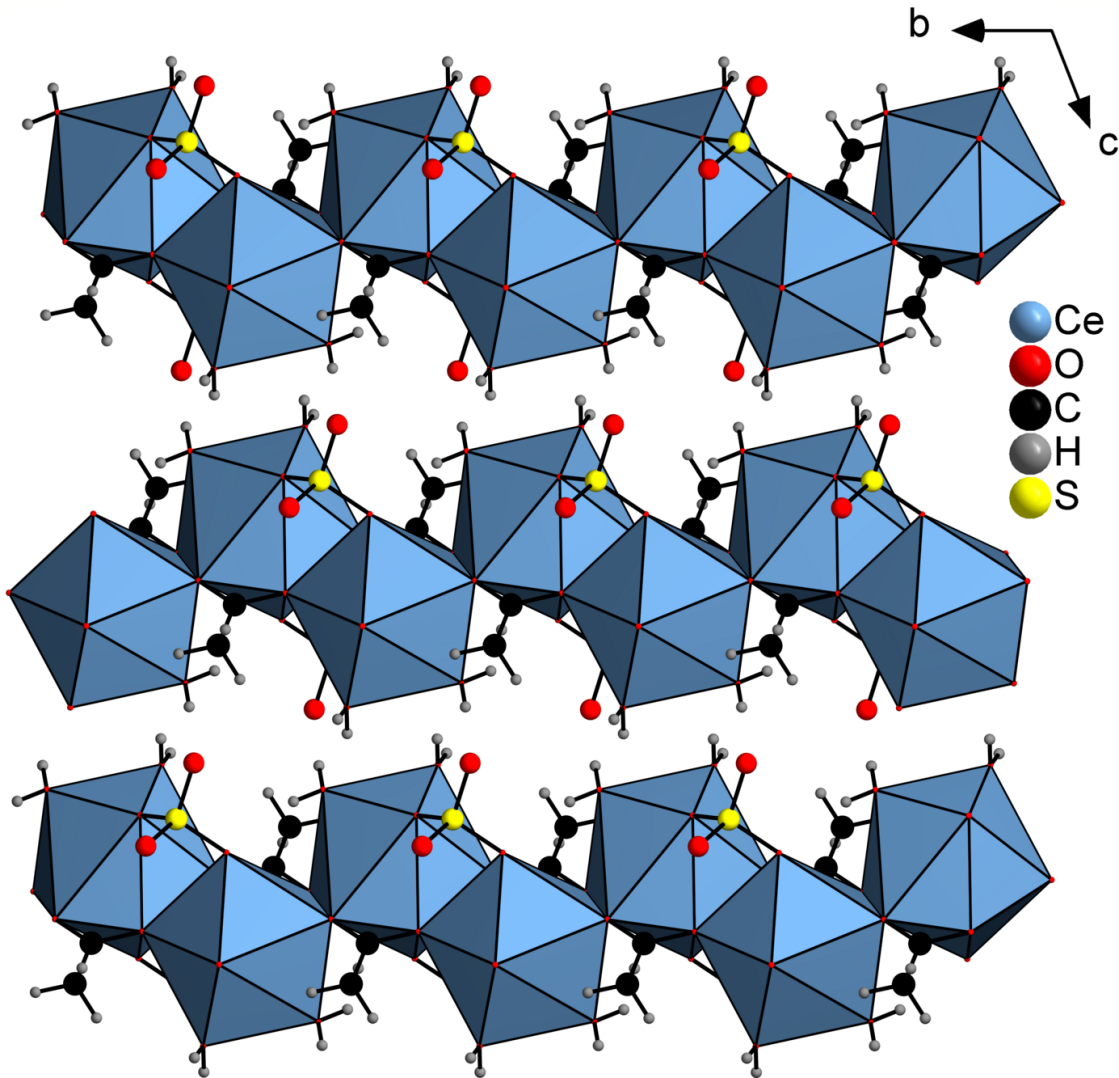
Crystal structure of the title compound viewed along the crystallographic *a* axis. The CeO_9_ units are displayed as polyhedra.

The asymmetric unit of the title compound, [Ce(CH_3_COO)(SO_4_)(H_2_O)_2_]_*n*_, consists of a crystallographically independent Ce^III^ cation, one acetate anion, one sulfate dianion and two crystallographically independent water mol­ecules, all in general positions (Fig. 1 ▸). Each Ce^III^ cation is ninefold coordinated by four O atoms of three symmetry-equivalent acetate anions, three O atoms of three symmetry-equivalent sulfate dianions and two crystallographically independent water mol­ecules. The Ce—O bond lengths range from 2.4385 (10) to 2.6518 (10) Å (Table 1 ▸) and the O—Ce—O angles reveal that the coordination geometry around the Ce^III^ ion is distorted. Two of the three acetate anions are coordinated with only one carboxyl O atom to the metal centres, whereas the third anion is coordinated with both O atoms to the Ce^III^ cations. Chains are formed by the acetate anions coordinated by three symmetry-equivalent Ce^III^ cations *via* the μ_3_-(*O*,*O*′,*O*,*O*′) bridging mode, which extend in the crystallographic *b*-axis direction (Fig. 2 ▸). The acetate C—O bond distances are almost the same (Table 1 ▸), indicating complete delocalization of the negative charge. The chains of cerium cations and acetate anions are linked by sulfate dianions to form layers lying parallel to the *ab* plane (Fig. 3 ▸). These layers are stacked along the crystallographic *c*-axis (Figs. 4 ▸ and 5 ▸).

## 3. Supra­molecular features

**Figure 6:**
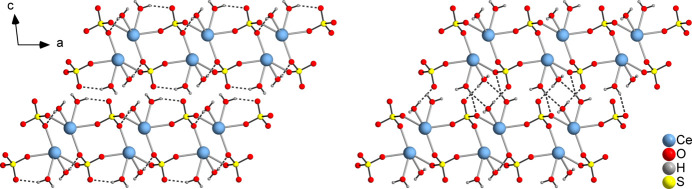
View along the layers with the intra­layer (left) and inter­layer (right) hydrogen bonds shown as dashed lines. The acetate anions were omitted for clarity.

Within the cerium–sulfate–acetate layers, intra­layer O—H⋯O hydrogen bonds (O7—H7*A*⋯O4, O8—H8*B*⋯O6) are observed between water H atoms (O7, O8) and sulfate O atoms (O4, O6) that are not involved in metal coordination (Fig. 6 ▸ left and Table 2 ▸). The same types of hydrogen bonds are also observed in the inter­connection of the layers by inter­layer O—H⋯O hydrogen bonds (O7—H7*B*⋯O6, O8—H8*A*⋯O5) between the H atoms of the water mol­ecules (O7, O8) and the O atoms (O6, O5) of the sulfate dianion. Also, inter­layer hydrogen bonding (O8—H8*A*⋯O7) between the two water mol­ecules is observed (Fig. 6 ▸ right). Most of the H⋯O distances are relatively short and the O—H⋯O angles are close to linear (Table 2 ▸), indicating that these are strong inter­actions (1.5–2.2 Å, 130–180°; Desiraju & Steiner, 1999 ▸). According to Table 2 ▸, the inter­layer hydrogen bonds O8—H8*A*⋯O5 and O8—H8*A*⋯O7 are rather weak. Two weak C—H⋯O inter­actions also occur. As a result of the inter­layer hydrogen bonding, a three-dimensional supra­molecular network is formed.

## 4. Database survey

A search for crystal structures containing any lanthanide, sulfate and acetate ions in the Cambridge Structural Database (CSD version 5.45, last update September 2024; Groom *et al.*, 2016 ▸) using CONQUEST (Bruno *et al.*, 2002 ▸) revealed six compounds with the composition [*Ln*(CH_3_COO)(SO_4_)(H_2_O)_2_]_*n*_ [*Ln* = La, Pr, Nd, Sm, Eu, CSD refcodes: EXURAR, EXURIZ, EXUREV, EXUROF, EXUQUK (Chen *et al.*, 2011 ▸) and *Ln* = Gd (FUSSIW; Liu *et al.*, 2009 ▸)], which are isostructural to the title compound. A search for cerium, sulfate and formate ions yielded two hits for a compound with the composition [Ce(HCOO)(SO_4_)(H_2_O)]_*n*_ (VESBOM, VESBOM01; Ju *et al.*, 2012 ▸), which is not isostructural to the title compound. This compound exhibits a chiral three-dimensional framework.

Searching for cerium(III) acetates (allowing the elements Ce, C, H, O), two compounds with three reported structures are encountered: [Ce(CH_3_COO)_3_(H_2_O)]_*n*_ (CEACET, CEACET01; Sadikov *et al.*, 1967 ▸; Junk *et al.*, 1999 ▸) and [Ce_2_(CH_3_COO)_6_(H_2_O)_2_]_*n*_·H_2_O (XECKEV; Junk *et al.*, 1999 ▸). In [Ce(CH_3_COO)_3_(H_2_O)]_*n*_, mono-periodic cerium acetate chains are present, which are inter­connected by hydrogen bonding between coordinating water mol­ecules and the acetate anions. [Ce_2_(CH_3_COO)_6_(H_2_O)_2_]_*n*_·H_2_O is build up by cerium acetate layers, which are bridged into a three-dimensional framework by hydrogen bonding between the water solvate mol­ecules and the acetate anions.

In the Inorganic Crystal Structure Database (ICSD release 2024.1; Zagorac *et al.*, 2019 ▸) twelve structures have been deposited for cerium(III) sulfates (composition: Ce^III^, S, O, H; number of elements: 4), namely Ce_2_(SO_4_)_3_(H_2_O)_9_ (ICSD-24184; Dereigne & Pannetier, 1968 ▸), Ce_2_(SO_4_)_3_(H_2_O)_8_ (ICSD-87633; Kepert *et al.*, 1999 ▸; ICSD-417418; Casari & Langer, 2007 ▸), Ce_2_(SO_4_)_3_(H_2_O)_5_ (ICSD-87635; Kepert *et al.*, 1999 ▸), Ce_2_(SO_4_)_3_(H_2_O)_4_ (ICSD-240937; Xu, 2008 ▸; ICSD-417417; Casari & Langer, 2007 ▸, ICSD-21073; Dereigne *et al.*, 1972 ▸), Ce(OH)(SO_4_) (ICSD-59922; Yang *et al.*, 2005 ▸), Ce(H_3_O)_0.5_(SO_4_)_1.5_(HSO_4_)_0.5_ (ICSD-414161; Yu *et al.*, 2004 ▸), Ce(HSO_4_)_3_ (ICSD-408961; Wickleder, 1998 ▸), (H_3_O)_0.444_Ce_0.888_(Ce_0.08_ (H_3_O)_0.14_)(SO_4_)_2_(H_2_O)_4.34_ (ICSD-92999; Filipenko *et al.*, 2001 ▸), (H_3_O)(Ce(SO_4_)_2_)(H_2_O) (ICSD-26559; Gatehouse & Pring, 1981 ▸). No crystal structure of anhydrous cerium(III) sulfate has been reported. Therefore, to the best of our knowledge, [Ce(CH_3_COO)(SO_4_)(H_2_O)_2_]_*n*_ is the first reported cerium acetate sulfate.

## 5. Synthesis and crystallization

**Figure 7:**
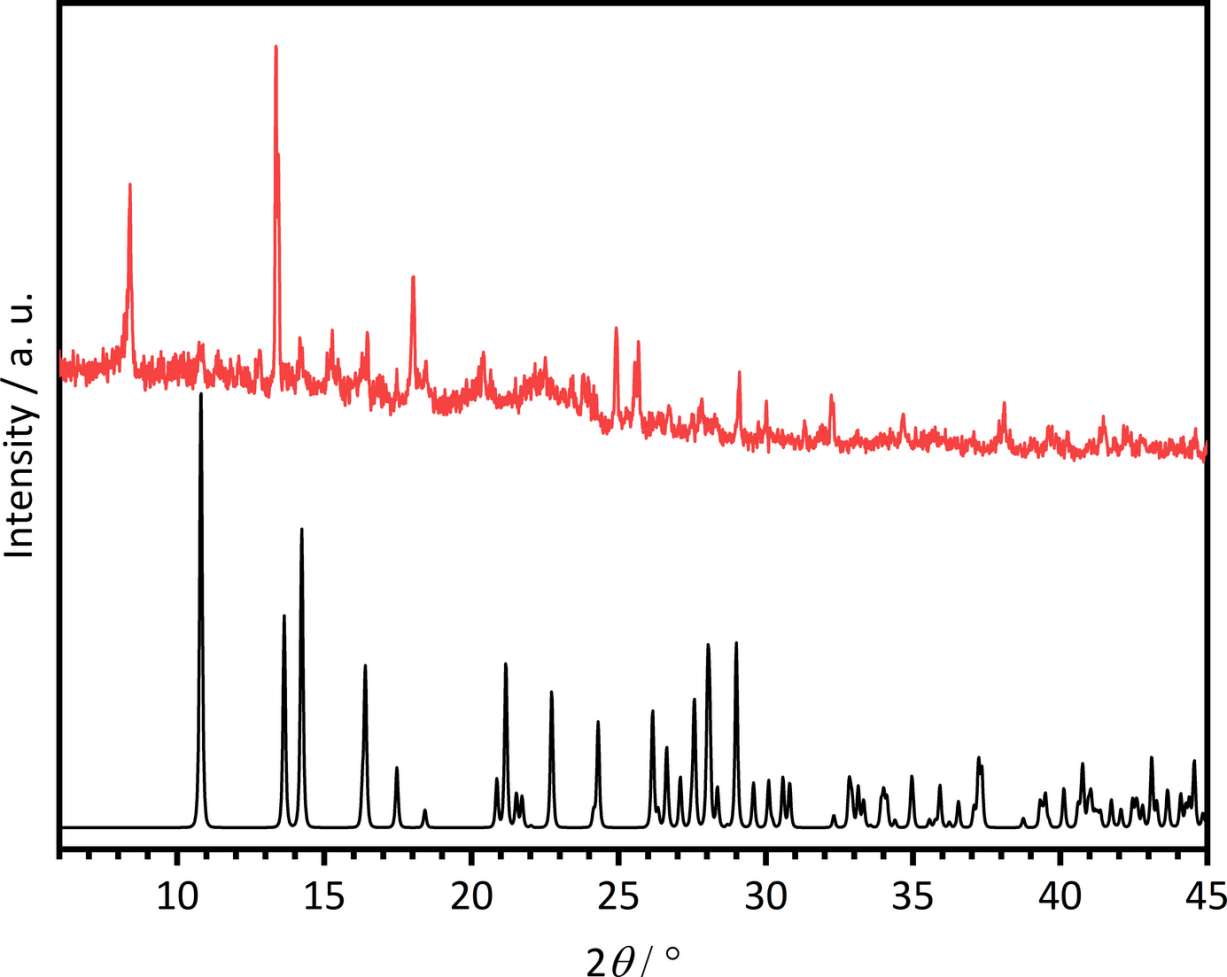
Experimental X-ray powder diffraction pattern of the batch from which the crystals were selected (red) and the calculated X-ray powder diffraction pattern of the title compound (black). The experimental pattern is very noisy because the yield was very low.

The synthesis was conducted applying the high-throughput method as described in the literature with our custom-made high-throughput setup (Radke *et al.* (2023 ▸). Single crystals of the title compound were serendipitously obtained by the reaction of 9.2 mg (0.053 mmol) of H_2_TDC, 400 µl (0.053 mmol) of a Ce(NO_3_)_3_·6H_2_O solution (*c* = 0.133 mol l^−1^) in H_2_O/EtOH (68:32), 365 µl of H_2_O/EtOH (68:32) and 235 µl of acetic acid in a 2 ml Teflon vial. The reactor was sealed and placed in a Memmert UFP400 oven heating the reaction mixture to 423 K over 24 h, holding that temperature for 192 h and afterwards slowly cooling down to room temperature over 48 h. The reaction mixture was filtered off and washed with H_2_O/EtOH (68:32) and dried under air. The product was obtained as a minor phase as indicated by powder X-ray diffraction (Fig. 7 ▸) but in the form of single crystals, which were suitable for single-crystal X-ray diffraction.

No sulfate-containing compounds were used consciously. Attempts to locate the source of the sulfate anions by testing the reactants and solvents for sulfate anions with aqueous BaCl_2_ for observing the formation of insoluble BaSO_4_ were unsuccessful. In an energy dispersive X-ray spectroscopy measurement of the used Ce(NO_3_)_3_·6H_2_O, no sulfur was found. Since the qu­antity of sulfate anions involved in the formation of the title compound seems to be untraceable, we find it most likely that the sulfate anions originate from leaching from the Teflon reactors, which were treated with per­oxy­monosulfuric acid prior to its use.

Attempts to prepare the title compound phase pure were unsuccessful. Conducting the described synthesis in a 7 ml pyrex tube for 6 h in absence of H_2_TDC, only an X-ray amorphous product was obtained. In addition, using equimolar amounts of Na_2_SO_4_ and Ce(NO_3_)_3_ only lead to the formation of an unknown crystalline compound. It should be noted that in the syntheses of [*Ln*(CH_3_COO)(SO_4_)(H_2_O)_2_]_*n*_ (*Ln* = La, Pr, Nd, Sm, Eu; Chen *et al.*, 2011 ▸), no sulfate source was used and the authors concluded that the sulfate dianions probably originate from the metal salts employed in the syntheses.

The powder X-ray diffraction pattern was collected on a Stoe Stadi P with a MYTHEN2 1K detector and Cu *K*α1 radiation.

## 6. Refinement

Crystal data, data collection and structure refinement details are summarized in Table 3 ▸. The C-bound H atoms were positioned with idealized geometry allowed to rotate but not to tip and were refined isotropically with *U*_iso_(H) = 1.5*U*_eq_(C) using a riding model. The O-bound H atoms were located in difference maps and were refined isotropically with varying coordinates.

## Supplementary Material

Crystal structure: contains datablock(s) I. DOI: 10.1107/S2056989024012313/hb8117sup1.cif

Structure factors: contains datablock(s) I. DOI: 10.1107/S2056989024012313/hb8117Isup2.hkl

CCDC reference: 2411959

Additional supporting information:  crystallographic information; 3D view; checkCIF report

## Figures and Tables

**Figure 5:**
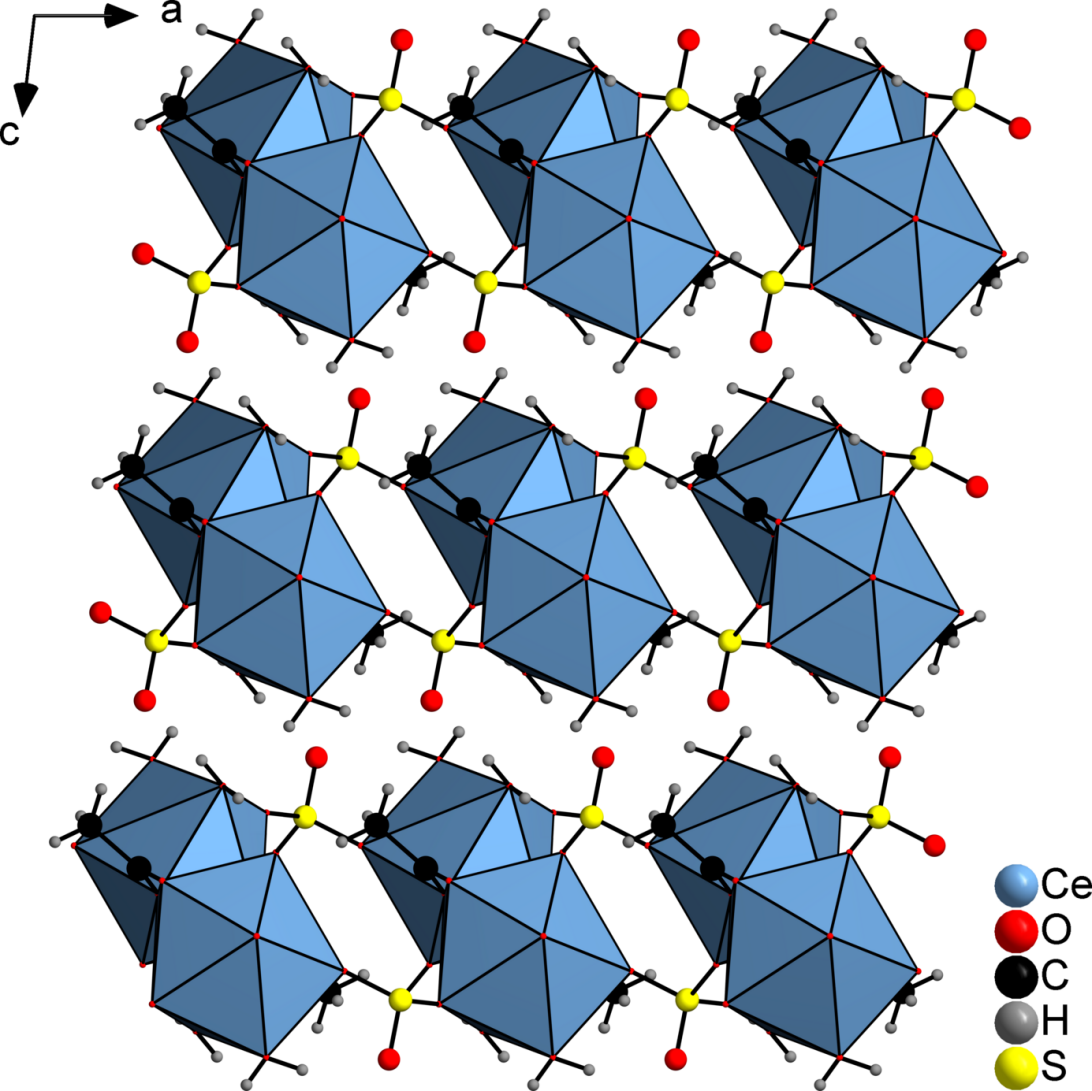
Crystal structure of the title compound viewed along the crystallographic *b* axis. The CeO_9_ units are displayed as polyhedra.

**Table 1:** Selected bond lengths (Å)

Ce1—O1	2.4608 (10)	Ce1—O5^iv^	2.4836 (10)
Ce1—O1^i^	2.6518 (10)	Ce1—O7	2.5678 (11)
Ce1—O2^ii^	2.5996 (10)	Ce1—O8	2.5127 (11)
Ce1—O2^i^	2.5992 (10)	C1—O1	1.2706 (16)
Ce1—O3	2.4385 (10)	C1—O2	1.2783 (17)
Ce1—O4^iii^	2.5119 (10)		

Symmetry codes: (i) 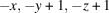; (ii) 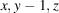; (iii) 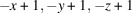; (iv) 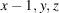.

**Table 2:** Hydrogen-bond geometry (Å, °)

*D*—H⋯*A*	*D*—H	H⋯*A*	*D*⋯*A*	*D*—H⋯*A*
O7—H7*A*⋯O4^v^	0.78 (3)	2.08 (3)	2.8400 (15)	165 (3)
O7—H7*B*⋯O6^vi^	0.79 (3)	1.95 (3)	2.7412 (15)	175 (3)
O8—H8*A*⋯O5^vi^	0.78 (3)	2.59 (3)	3.1389 (15)	129 (2)
O8—H8*A*⋯O7^vii^	0.78 (3)	2.30 (3)	3.0386 (16)	158 (2)
O8—H8*B*⋯O6	0.87 (2)	1.99 (2)	2.7976 (15)	154 (2)
C2—H2*A*⋯O4	0.98	2.43	3.3245 (18)	152
C2—H2*C*⋯O3^viii^	0.98	2.40	3.2909 (18)	151

Symmetry codes: (v) 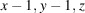; (vi) 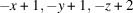; (vii) 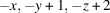; (viii) 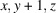.

**Table 3:** Experimental details

Crystal data	
Chemical formula	[Ce(C_2_H_3_O_2_)(SO_4_)(H_2_O)_2_]
*M*_r_	331.26
Crystal system, space group	Triclinic, *P* 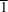
Temperature (K)	100
*a*, *b*, *c* (Å)	6.8424 (1), 6.9984 (1), 8.7888 (2)
α, β, γ (°)	110.448 (2), 90.099 (2), 107.307 (2)
*V* (Å^3^)	373.85 (1)
*Z*	2
Radiation type	Mo *K*α
μ (mm^−1^)	6.38
Crystal size (mm)	0.08 × 0.08 × 0.03

Data collection	
Diffractometer	XtaLAB Synergy, Dualflex, HyPix
Absorption correction	Multi-scan (CrysAlisPr; Rigaku OD, 2023 ▸)
*T*_min_, *T*_max_	0.243, 0.264
No. of measured, independent and observed [*I* > 2σ(*I*)] reflections	8752, 2085, 2066
*R*_int_	0.014
(sin θ/λ)_max_ (Å^−1^)	0.708

Refinement	
*R*[*F*^2^ > 2σ(*F*^2^)], *wR*(*F*^2^), *S*	0.010, 0.025, 1.08
No. of reflections	2085
No. of parameters	126
H-atom treatment	H atoms treated by a mixture of independent and constrained refinement
Δρ_max_, Δρ_min_ (e Å^−3^)	0.75, −0.40

Computer programs: *CrysAlis PRO* (Rigaku OD, 2023 ▸), *SHELXT2014/4* (Sheldrick, 2015*a* ▸), *SHELXL2016/6* (Sheldrick, 2015*b* ▸), *DIAMOND* (Brandenburg & Putz, 1999 ▸), *XP* in *SHELXTL-PC* (Sheldrick, 2008 ▸) and *publCIF* (Westrip, 2010 ▸).

## supplementary crystallographic information

### Poly[µ3-acetato-diaqua-µ3-sulfato-cerium(III)] Crystal data

**Table:**

[Ce(C_2_H_3_O_2_)(SO_4_)(H_2_O)_2_]	*Z* = 2
*M**_r_* = 331.26	*F*(000) = 314
Triclinic, *P*1	*D*_x_ = 2.943 Mg m^−^^3^
*a* = 6.8424 (1) Å	Mo *K*α radiation, λ = 0.71073 Å
*b* = 6.9984 (1) Å	Cell parameters from 7869 reflections
*c* = 8.7888 (2) Å	θ = 2.5–30.2°
α = 110.448 (2)°	µ = 6.38 mm^−^^1^
β = 90.099 (2)°	*T* = 100 K
γ = 107.307 (2)°	Plate, colorless
*V* = 373.85 (1) Å^3^	0.08 × 0.08 × 0.03 mm

### Poly[µ3-acetato-diaqua-µ3-sulfato-cerium(III)] Data collection

**Table:**

XtaLAB Synergy, Dualflex, HyPix diffractometer	2085 independent reflections
Radiation source: micro-focus sealed X-ray tube, PhotonJet (Mo) X-ray Source	2066 reflections with *I* > 2σ(*I*)
Mirror monochromator	*R*_int_ = 0.014
Detector resolution: 10.0000 pixels mm^-1^	θ_max_ = 30.2°, θ_min_ = 2.5°
ω scans	*h* = −9→9
Absorption correction: multi-scan (CrysAlisPr; Rigaku OD, 2023)	*k* = −9→9
*T*_min_ = 0.243, *T*_max_ = 0.264	*l* = −12→12
8752 measured reflections	

### Poly[µ3-acetato-diaqua-µ3-sulfato-cerium(III)] Refinement

**Table:**

Refinement on *F*^2^	Primary atom site location: dual
Least-squares matrix: full	Hydrogen site location: mixed
*R*[*F*^2^ > 2σ(*F*^2^)] = 0.010	H atoms treated by a mixture of independent and constrained refinement
*wR*(*F*^2^) = 0.025	*w* = 1/[σ^2^(*F*_o_^2^) + (0.0141*P*)^2^ + 0.1685*P*] where *P* = (*F*_o_^2^ + 2*F*_c_^2^)/3
*S* = 1.08	(Δ/σ)_max_ = 0.001
2085 reflections	Δρ_max_ = 0.75 e Å^−^^3^
126 parameters	Δρ_min_ = −0.40 e Å^−^^3^
0 restraints	

### Poly[µ3-acetato-diaqua-µ3-sulfato-cerium(III)] Special details

**Table:**

Geometry. All esds (except the esd in the dihedral angle between two l.s. planes) are estimated using the full covariance matrix. The cell esds are taken into account individually in the estimation of esds in distances, angles and torsion angles; correlations between esds in cell parameters are only used when they are defined by crystal symmetry. An approximate (isotropic) treatment of cell esds is used for estimating esds involving l.s. planes.

### Poly[µ3-acetato-diaqua-µ3-sulfato-cerium(III)] Fractional atomic coordinates and isotropic or equivalent isotropic displacement parameters (Å^2^)

**Table:**

	*x*	*y*	*z*	*U*_iso_*/*U*_eq_	
Ce1	0.12292 (2)	0.35141 (2)	0.63283 (2)	0.00574 (3)	
O1	0.17608 (15)	0.68328 (15)	0.57726 (12)	0.00892 (18)	
O2	0.18457 (16)	0.98045 (16)	0.54087 (12)	0.00910 (18)	
C1	0.2619 (2)	0.8836 (2)	0.61166 (17)	0.0078 (2)	
C2	0.4512 (2)	1.0007 (2)	0.73196 (19)	0.0123 (3)	
H2A	0.565935	0.952456	0.685357	0.015*	
H2B	0.426361	0.970895	0.832451	0.015*	
H2C	0.485570	1.155597	0.757098	0.015*	
S1	0.70445 (5)	0.60045 (5)	0.75494 (4)	0.00687 (6)	
O3	0.49759 (15)	0.45194 (16)	0.67514 (13)	0.00991 (19)	
O4	0.79111 (16)	0.73412 (16)	0.65621 (12)	0.00951 (19)	
O5	0.84036 (15)	0.47553 (16)	0.76662 (12)	0.00945 (18)	
O6	0.68899 (16)	0.74388 (17)	0.92067 (12)	0.01042 (19)	
O7	0.00409 (18)	0.17358 (18)	0.84206 (14)	0.0114 (2)	
H7A	−0.055 (4)	0.050 (4)	0.807 (3)	0.034 (7)*	
H7B	0.088 (5)	0.193 (5)	0.913 (4)	0.044 (8)*	
O8	0.26010 (17)	0.62451 (18)	0.91554 (13)	0.0125 (2)	
H8A	0.203 (4)	0.659 (4)	0.992 (3)	0.028 (6)*	
H8B	0.391 (4)	0.673 (4)	0.950 (3)	0.023 (6)*	

### Poly[µ3-acetato-diaqua-µ3-sulfato-cerium(III)] Atomic displacement parameters (Å^2^)

**Table:**

	*U*^11^	*U*^22^	*U*^33^	*U*^12^	*U*^13^	*U*^23^
Ce1	0.00598 (4)	0.00511 (4)	0.00627 (4)	0.00165 (3)	0.00012 (3)	0.00234 (3)
O1	0.0103 (5)	0.0069 (4)	0.0098 (5)	0.0026 (4)	0.0007 (4)	0.0036 (4)
O2	0.0097 (4)	0.0078 (4)	0.0107 (5)	0.0032 (4)	−0.0004 (4)	0.0041 (4)
C1	0.0073 (6)	0.0083 (6)	0.0078 (6)	0.0031 (5)	0.0025 (5)	0.0026 (5)
C2	0.0118 (6)	0.0102 (6)	0.0137 (7)	0.0021 (5)	−0.0033 (5)	0.0043 (5)
S1	0.00646 (14)	0.00716 (14)	0.00742 (14)	0.00237 (11)	0.00052 (11)	0.00302 (11)
O3	0.0067 (4)	0.0099 (4)	0.0115 (5)	0.0014 (4)	−0.0003 (4)	0.0030 (4)
O4	0.0114 (5)	0.0088 (4)	0.0098 (5)	0.0032 (4)	0.0025 (4)	0.0050 (4)
O5	0.0088 (4)	0.0111 (5)	0.0113 (5)	0.0055 (4)	0.0012 (4)	0.0054 (4)
O6	0.0107 (5)	0.0121 (5)	0.0078 (5)	0.0044 (4)	0.0012 (4)	0.0024 (4)
O7	0.0135 (5)	0.0087 (5)	0.0111 (5)	0.0014 (4)	−0.0022 (4)	0.0044 (4)
O8	0.0094 (5)	0.0153 (5)	0.0085 (5)	0.0018 (4)	0.0009 (4)	0.0012 (4)

### Poly[µ3-acetato-diaqua-µ3-sulfato-cerium(III)] Geometric parameters (Å, º)

**Table:**

Ce1—O1	2.4608 (10)	C1—C2	1.4916 (19)
Ce1—O1^i^	2.6518 (10)	C2—H2A	0.9800
Ce1—O2^ii^	2.5996 (10)	C2—H2B	0.9800
Ce1—O2^i^	2.5992 (10)	C2—H2C	0.9800
Ce1—O3	2.4385 (10)	S1—O3	1.4790 (10)
Ce1—O4^iii^	2.5119 (10)	S1—O4	1.4895 (10)
Ce1—O5^iv^	2.4836 (10)	S1—O5	1.4784 (10)
Ce1—O7	2.5678 (11)	S1—O6	1.4773 (11)
Ce1—O8	2.5127 (11)	O7—H7A	0.78 (3)
Ce1—C1^i^	3.0316 (14)	O7—H7B	0.79 (3)
C1—O1	1.2706 (16)	O8—H8A	0.78 (3)
C1—O2	1.2783 (17)	O8—H8B	0.87 (2)
			
O1—Ce1—O1^i^	67.57 (4)	O7—Ce1—C1^i^	97.45 (4)
O1—Ce1—O2^ii^	145.60 (3)	O8—Ce1—O1^i^	130.03 (3)
O1—Ce1—O2^i^	116.63 (3)	O8—Ce1—O2^ii^	120.31 (3)
O1^i^—Ce1—C1^i^	24.69 (3)	O8—Ce1—O2^i^	144.47 (3)
O1—Ce1—C1^i^	92.06 (3)	O8—Ce1—C1^i^	143.46 (4)
O1—Ce1—O4^iii^	74.19 (3)	O8—Ce1—O7	71.10 (4)
O1—Ce1—O5^iv^	78.93 (3)	Ce1—O1—Ce1^i^	112.43 (4)
O1—Ce1—O7	143.73 (3)	C1—O1—Ce1	151.89 (9)
O1—Ce1—O8	80.50 (3)	C1—O1—Ce1^i^	94.63 (8)
O2^ii^—Ce1—O1^i^	107.17 (3)	Ce1^i^—O2—Ce1^v^	116.36 (4)
O2^i^—Ce1—O1^i^	49.44 (3)	C1—O2—Ce1^v^	134.95 (9)
O2^i^—Ce1—O2^ii^	63.64 (4)	C1—O2—Ce1^i^	96.92 (8)
O2^i^—Ce1—C1^i^	24.75 (3)	O1—C1—Ce1^i^	60.68 (7)
O2^ii^—Ce1—C1^i^	85.25 (3)	O1—C1—O2	119.01 (12)
O3—Ce1—O1	86.58 (3)	O1—C1—C2	119.73 (12)
O3—Ce1—O1^i^	140.56 (3)	O2—C1—Ce1^i^	58.33 (7)
O3—Ce1—O2^ii^	77.38 (3)	O2—C1—C2	121.26 (12)
O3—Ce1—O2^i^	137.85 (3)	C2—C1—Ce1^i^	179.59 (10)
O3—Ce1—C1^i^	146.27 (3)	C1—C2—H2A	109.5
O3—Ce1—O4^iii^	78.81 (3)	C1—C2—H2B	109.5
O3—Ce1—O5^iv^	139.02 (3)	C1—C2—H2C	109.5
O3—Ce1—O7	103.44 (4)	H2A—C2—H2B	109.5
O3—Ce1—O8	69.46 (4)	H2A—C2—H2C	109.5
O4^iii^—Ce1—O1^i^	66.07 (3)	H2B—C2—H2C	109.5
O4^iii^—Ce1—O2^i^	75.14 (3)	O3—S1—O4	108.88 (6)
O4^iii^—Ce1—O2^ii^	72.95 (3)	O5—S1—O3	109.66 (6)
O4^iii^—Ce1—C1^i^	68.47 (3)	O5—S1—O4	110.12 (6)
O4^iii^—Ce1—O7	141.63 (3)	O6—S1—O3	109.99 (6)
O4^iii^—Ce1—O8	140.26 (4)	O6—S1—O4	108.79 (6)
O5^iv^—Ce1—O1^i^	66.55 (3)	O6—S1—O5	109.39 (6)
O5^iv^—Ce1—O2^ii^	131.94 (3)	S1—O3—Ce1	153.24 (6)
O5^iv^—Ce1—O2^i^	82.17 (3)	S1—O4—Ce1^iii^	133.75 (6)
O5^iv^—Ce1—C1^i^	73.04 (3)	S1—O5—Ce1^vi^	140.99 (6)
O5^iv^—Ce1—O4^iii^	131.50 (3)	Ce1—O7—H7A	116.9 (19)
O5^iv^—Ce1—O7	70.70 (3)	Ce1—O7—H7B	118 (2)
O5^iv^—Ce1—O8	70.42 (4)	H7A—O7—H7B	106 (3)
O7—Ce1—O1^i^	115.10 (3)	Ce1—O8—H8A	129.9 (18)
O7—Ce1—O2^ii^	70.36 (3)	Ce1—O8—H8B	121.1 (15)
O7—Ce1—O2^i^	78.89 (3)	H8A—O8—H8B	107 (2)
			
Ce1—O1—C1—Ce1^i^	−164.6 (2)	O3—S1—O4—Ce1^iii^	−60.31 (9)
Ce1—O1—C1—O2	−165.26 (13)	O3—S1—O5—Ce1^vi^	102.11 (10)
Ce1^i^—O1—C1—O2	−0.66 (13)	O4—S1—O3—Ce1	−92.36 (15)
Ce1^i^—O1—C1—C2	179.93 (11)	O4—S1—O5—Ce1^vi^	−17.68 (11)
Ce1—O1—C1—C2	15.3 (3)	O5—S1—O3—Ce1	147.09 (13)
Ce1^v^—O2—C1—Ce1^i^	138.87 (12)	O5—S1—O4—Ce1^iii^	59.96 (9)
Ce1^i^—O2—C1—O1	0.68 (13)	O6—S1—O3—Ce1	26.76 (16)
Ce1^v^—O2—C1—O1	139.54 (11)	O6—S1—O4—Ce1^iii^	179.83 (7)
Ce1^i^—O2—C1—C2	−179.92 (11)	O6—S1—O5—Ce1^vi^	−137.19 (9)
Ce1^v^—O2—C1—C2	−41.1 (2)		

Symmetry codes: (i) −*x*, −*y*+1, −*z*+1; (ii) *x*, *y*−1, *z*; (iii) −*x*+1, −*y*+1, −*z*+1; (iv) *x*−1, *y*, *z*; (v) *x*, *y*+1, *z*; (vi) *x*+1, *y*, *z*.

### Poly[µ3-acetato-diaqua-µ3-sulfato-cerium(III)] Hydrogen-bond geometry (Å, º)

**Table:**

*D*—H···*A*	*D*—H	H···*A*	*D*···*A*	*D*—H···*A*
O7—H7*A*···O4^vii^	0.78 (3)	2.08 (3)	2.8400 (15)	165 (3)
O7—H7*B*···O6^viii^	0.79 (3)	1.95 (3)	2.7412 (15)	175 (3)
O8—H8*A*···O5^viii^	0.78 (3)	2.59 (3)	3.1389 (15)	129 (2)
O8—H8*A*···O7^ix^	0.78 (3)	2.30 (3)	3.0386 (16)	158 (2)
O8—H8*B*···O6	0.87 (2)	1.99 (2)	2.7976 (15)	154 (2)
C2—H2*A*···O4	0.98	2.43	3.3245 (18)	152
C2—H2*C*···O3^v^	0.98	2.40	3.2909 (18)	151

Symmetry codes: (v) *x*, *y*+1, *z*; (vii) *x*−1, *y*−1, *z*; (viii) −*x*+1, −*y*+1, −*z*+2; (ix) −*x*, −*y*+1, −*z*+2.
